# Patients’ perception of lifestyle advice as a mechanism between health shocks and health behaviours: Evidence from a longitudinal study in China

**DOI:** 10.7189/jogh.14.04059

**Published:** 2024-03-22

**Authors:** Peng Zhang, Luying Zhang, Wen Chen

**Affiliations:** 1 School of Public Health, Fudan University, Xuhui District, Shanghai, China; 2 School of Humanities, Shanghai Institute of Technology, Fengxian District, Shanghai, China

## Abstract

**Background:**

The heavy burden of non-communicable chronic diseases (NCDs) and the deficiency of health behaviours (HB) are threatening the middle- and older-aged population in China. However, little is known about the relational mechanism between health shocks (HS) and HBs, while the importance of patients’ perception of lifestyle advice (PPLA) to initiate HB is insufficiently emphasised. In this study, we aimed to examine this perception as a mediator between HS and HB and the variety of mediation effects caused by the different contents of lifestyle advice.

**Methods:**

We retrieved panel data from the two latest waves of a nationally representative cohort, the China Health and Retirement Longitudinal Study (CHARLS). After constructing well-balanced groups through propensity score matching, we conducted descriptive and multilevel logistic regression analyses to analyse the state of and factors influencing PPLA. We employed the Preacher’s Sobel test with 1000 replications bootstrap to examine the mediating effect of PPLA.

**Results:**

On a sample of 7922 respondents (post-propensity score matching), we found that HSs had a positive direct effect on HB, with observed decreases in smoking and drinking and increases in exercise. A limited and selected perception of lifestyle advice showed a gap between the advice given by providers and perceived by patients, with gender, education level, social support inside the family, self-reported health, comorbidity, treatment regimens, and utilisation of family doctors being significant influencing factors. Nevertheless, any content of lifestyle advice partially mediated the relationship in which HS increases non-addictive HB (exercise), while only the targeted and detailed content of lifestyle advice about corresponding behaviours partially mediated the effect between HS and addictive HB (smoking and drinking).

**Conclusions:**

This study provides the first evidence that PPLA partially mediates the positive effect of HS on HB. Personalised chronic disease management; targeted advice and interventions; and multiple social resources COULD BE beneficial for patients with HS to initiate HB.

The burden of non-communicable chronic diseases (NCDs) is a major health concern in China, with an exceedingly high, rapidly increasing prevalence among the middle- and older-aged Chinese population [1]. However, health behaviours (HB), which are important in managing NCDs, are insufficiently present among this at-risk age group [2]. In the World Health Organization’s 2020 Global Tobacco Consumption Report, Chinese residents had a smoking rate of approximately 25.3%, significantly higher compared to other countries [3]. The average alcohol consumption among the Chinese population also remained higher than the global average, with a substantial increase of 76% from 2005 to 2016 [4]. Furthermore, according to the 2020 National Fitness Activity Survey Report published by the General Administration of Sport of China, the proportion of physical activity among middle-aged and elderly residents decreases with age and less than 25% of them had regular physical activity, defined as weekly exercising three or more time [5].

HB are complex and influenced by various factors, many of which are stable and resistant to change [6,7]. However, health shocks (HS), characterised as abrupt declines in health resulting from illnesses or accidents [8], have been found to positively influence health behaviours, including by reducing smoking prevalence and daily cigarette consumption [9–14]; controlling alcohol consumption and changing drinking habits [11,12]; and increasing the probability of exercising [12]. Similarly, our previous study conducted among the Chinese population confirmed the positive impact of HS on changes in HB [14].

Yet despite this evidence, the possible mechanism behind this relationship has only been discussed rather than empirically examined, with hypotheses that HS affects HB by triggering individuals’ health concerns [15,16]; by individuals gaining extra social support after HS [17–19]; and by the HS providing a moment in which health care providers offer advice and interventions [17,18,20–22]. Meanwhile, patients’ perception of lifestyle advice (PPLA) has emerged as the most extensively discussed and potentially relevant mechanism, with existing research evidence [9–13,20]. Previous studies demonstrated that HS promote PPLA because patients used health care services for medical consultations following HS, thus providing an opportunity for health education by physicians [11,12,17–22] and by patients showing increased health awareness and heightened receptiveness to receiving lifestyle advice [23–25]. Other studies linked PPLA and HB by proving that patients who are more inclined to accept comprehensive and tailored lifestyle advice are more likely to make changes in HB by reducing smoking [23,24,26] or alcohol consumption [27] and increasing exercise [28–30].

Current literature is limited in several ways. For example, the mechanism of the relationship between HS and HB was not examined, while the role of health care providers in the process remains poorly understood. Despite the plausible associations between HS, PPLA, and HB, they have mostly been studied separately with cross-sectional designs, leading to calls for longitudinal analyses. Furthermore, although the potential mediation role of PPLA is known, it remains unclear how to effectively provide lifestyle advice during the educational opportunity brought by a HS in order to enhance patient acceptance and engagement in adopting HB.

To address these gaps, we retrieved panel data from the China Health and Retirement Longitudinal Study (CHARLS) and constructed several models to describe the current state, analyse the influencing factors, and examine the mediating effect of PPLA in the relationship between HS and HB. We thereby hypothesised that PPLA serves as a mediating mechanism between HS and HB.

## METHODS

### Conceptual framework

**Figure 1:**
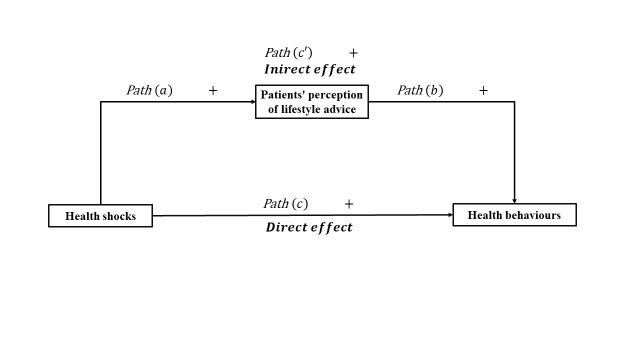
Conceptual framework of PPLA as a mechanism between HS and HB.

Our conceptual framework (**Figure 1**), based on former studies and the traditional mediator proposed by Baron and Kenny [31], attempted to illustrate a possible mediating path to examine PPLA as a mechanism between HS and HB. Within it, we employed a four-component empirical analysis, where HS directly affects HB (path C); HS increases the likelihood of patients perceiving lifestyle advice (path A); PPLA positively influences personal HB (path B); and HS indirectly affects HB through PPLA (path C’).

### Study design

CHARLS is a nationally representative cohort study of middle- and older-aged Chinese individuals aged ≥45 years, conducted in waves from 2011 onwards based on random multistage probability-proportional-to-size sampling from 28 provinces in mainland China. The latest follow-up study in 2018 included data on 19 816 respondents (e.g. demographics, employment, economics, health status, and health care insurance). A response rate of >86% and a follow-up rate of the baseline sample of >70% suggest high reliability and validity.

### Study population

**Figure 2:**
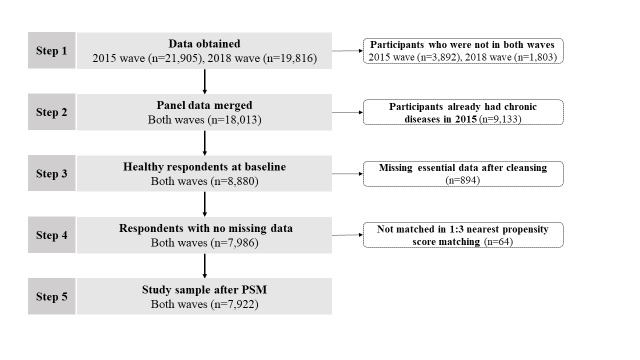
Flowchart of study participants.

Since more detailed information relating to HB became available after 2015, we extracted data from the 2015–18 waves. Following cleaning, we merged the balanced longitudinal data and selected individuals: who participated in both the 2015 and 2018 wave; who were not diagnosed with chronic diseases in 2015 (including hypertension, diabetes, cancer, heart disease, stroke, lung disease, etc.); who had no missing data relating to essential measurements; and who were matched by propensity score matching (PSM) to gain a more comparative characteristic at baseline. The final sample comprised 7922 respondents (**Figure 2**).

### Measurements

#### Health shocks

We selected HS as the independent variable, measured by the new diagnosis of chronic diseases, which is the most common measurement to reflect the suddenness and exogeneity of HS [32,33]. Specifically, we used discrete indicators of hypertension HS and diabetes HS based on responses to the question of whether the individuals had no pre-existing chronic diseases in 2015 and were newly diagnosed with hypertension or diabetes in 2018 (‘yes’ for 1 if HS occurred, ‘no’ for 0 if not). We focussed on these two conditions because they result in a significant disease burden [1], but also because HB exert a more significant influence on patients with these relatively long-term and not immediately life-threatening HS. Our previous study has also demonstrated the substantial impact of these two HS on various HB [14], and health care providers are obligated to provide lifestyle advice to patients and record it in the information system during each health care encounter according to clinical guidelines and disease management protocols for hypertension and diabetes [34,35], thereby providing a base for investigating the underlying mechanism regarding PPLA.

#### Health behaviours

The dependent variables were three HB, with their differences being compared before HS in 2015 and after HS in 2018. We coded binomial variables of smoking by the questions about smoking cigarettes, cigars, etc. (1 for smoking, 0 for non-smoking); of drinking based on the questions about drinking any alcoholic beverages (1 for drinking, 0 for non-drinking); and for exercise by combining participation in activities and whether with the purpose of exercise (1 for exercising, 0 for not exercising).

#### Patients’ perception of lifestyle advice

We coded four binomial variables of the mediating factor, and PPLA by the question of whether the respondent perceived and self-reported advice by health providers to take HB after HS and what the contents of lifestyle advice are (smoking cessation, dietary, or exercise). The importance of patient-perceived advice rather than the provision of advice determined the success of turning interventions into HB [36,37], so we chose PPLA as a mediator to reflect whether the HS group accepted, comprehended, and received lifestyle advice.

#### Covariates

We included other covariates to describe characteristics, analyse the influencing factors, and control in models. The ordered variables included time, age, sex, belonging to Han population, marital status, education level, occupation, residence, health insurance type, household expenditure, self-reported health status, regional location, provincial gross domestic product, treatment regimens, comorbidity, health care utilisation, and satisfaction with health care. The numerical variables were social support inside the family (measured by the number of household members who live together with the respondents [38]); social support outside the family (measured by the number of social activities the respondents participated in the last month [39]); and activities of daily living (scores of ADLs range from 6–24 [40]).

### Statistical analysis

First, we applied PSM to match the characteristics between HS and non-HS groups, selecting 1:3 nearest matching as the most effective method to form a more comparative pair. Then, we described and explored the sample characteristics in both HS and non-HS groups at baseline. The results of the reduced bias and the *P*-value after 1:3 nearest matching showed no statistical difference between the groups, suggesting they were well-balanced.

We then conducted multilevel logistic regression analyses, which were suitable for panel data with a multilayer nested structure from the nationally sampled survey with the repeated measure design [41]. Specifically, we conducted logit models with three levels (individual-wave-level, individual-level, and community-level) to capture the clustered variations and estimate the multilevel random intercepts [42], gradually including the influencing factors in the models, as follows:

− Model 1: We included the personal factors and environmental factors and controlled for three levels;
− Model 2: We added factors related to dealing with HS or having other HS;
− Model 3: We added factors related to healthcare.

Lastly, based on the product-of-coefficients approach to estimate the mediating effect proposed by Baron and Kenny [31], we used Preacher’s Sobel test with 1000 replications bootstrap to examine the mediating effect of PPLA between HS and HB, designed to test a multi-level mediation effect with non-normality distribution [43,44]. We considered a *P*-value <0.05 as statistically significant.

## RESULTS

### Health shocks and participant characteristics

During 2015–2018, HS occurred in 2741 (31.19%) of 7922 respondents (hypertension HS: 1829, diabetes HS: 852, both hypertension and diabetes HS: 210). The HS group tended to be elderly, not married, less educated, retired, and with slightly worse health status; after PSM, these characteristics were not statistically different at baseline (**Table 1**).

**Table 1:** Descriptive table of the study sample after 1:3 nearest matching at baseline*

Categorical variables	HS (n = 2471), n (%)	Non-HS (n = 5451), n (%)
Age		
*45–59*	1144 (46.30)	3380 (62.019)
*60–74*	1120 (45.33)	1798 (32.98)
*75–89*	205 (8.30)	264 (4.84)
*≥90*	2 (0.08)	9 (0.17)
Sex		
*Male*	1219 (49.33)	2691 (49.37)
*Female*	1252 (50.67)	2760 (50.63)
Ethnic groups		
*Han population*	2280 (92.27)	5129 (94.09)
*Non-Han population*	191 (7.73)	322 (5.91)
Married		
*Yes*	2177 (88.10)	4955 (90.90)
*No*	294 (11.90)	496 (9.10)
Education level		
*No formal education*	1118 (45.24)	2245 (41.19)
*Elementary school*	570 (23.07)	1207 (22.14)
*Secondary school*	508 (20.56)	1336 (24.51)
*High school*	236 (9.55)	583 (10.70)
*College and above*	39 (1.58)	80 (1.47)
Occupation		
*Others*	238 (9.63)	414 (7.59)
*Farmer*	1829 (74.02)	4336 (79.55)
*Civil servant*	91 (3.68)	209 (3.83)
*Retired*	313 (12.67)	492 (9.03)
Residence		
*Rural*	1947 (78.79)	4567 (83.78)
*Urban*	524 (21.21)	884 (16.22)
Health insurance type		
*No insurance*	62 (2.51)	151 (2.77)
*URBMI*	2031 (82.19)	4636 (85.05)
*UEMI*	378 (15.30)	664 (12.18)
Household expenditure		
*Lowest quantile*	756 (39.59)	1622 (29.76)
*Middle quantile*	883 (35.73)	1902 (34.89)
*Highest quantile*	832 (33.67)	1927 (35.35)
Self-reported health status		
*Lowest level*	1326 (53.66)	2209 (40.52)
*Middle level*	768 (31.08)	1852 (33.98)
*Highest level*	377 (15.26)	1390 (25.50)
Regional location		
*East area*	843 (34.12)	1929 (35.39)
*Mid area*	833 (33.71)	1770 (32.47)
*West area*	795 (32.17)	1752 (32.14)
Provincial GDP		
*Lowest quantile*	900 (36.42)	1998 (36.65)
*Middle quantile*	752 (30.43)	1628 (29.87)
*Highest quantile*	819 (33.14)	1825 (33.48)
Numerical variables, x̄ (SD)		
*Social support inside the family*	1.76 (1.92)	1.77 (1.97)
*Social support outside the family*	1.98 (1.23)	1.06 (1.27)
Activities of daily living, x̄ (SD)	6.95 (1.93)	6.30 (1.48)

GDP – gross domestic product, HS – health shocks, SD – standard deviation, NRCMS – New Rural Cooperative Medical Scheme, UEMI – Urban Employee Basic Medical Insurance, URBMI – Urban Resident Basic Medical Insurance, x̄ – mean

*Hypertension HS: n = 1829, diabetes HS: n = 852.

### PPLA and its influencing factors

More than half of individuals with HS reported receiving lifestyle advice from health care providers, with similar results for both HS groups (hypertension HS: 55.33%, diabetes HS: 66.43%).

The different contents of advice were also received by patients differently. Dietary advice was the most common and acceptable content, with 45.54% of the hypertension HS and 61.97% of the diabetes HS perceived. Physical activity advice followed, with smoking cessation advice perceived by the lowest proportion (hypertension HS: 27.78%, diabetes HS: 28.76%) in both groups (**Table 2**).

**Table 2:** PPLA after hypertension HS and diabetes HS

PPLA	Hypertension HS, n (%)	Diabetes HS, n (%)
Total	1829 (100.00)	852 (100.00)
Non-perceived advice	817 (44.67)	286 (33.57)
Perceived advice	1012 (55.33)	566 (66.43)
*Smoking cessation advice*	508 (27.78)	245 (28.76)
*Dietary advice*	833 (45.54)	528 (61.97)
*Physical activity advice*	698 (38.16)	409 (48.00)

HS – health shocks, PPLA – patients’ perception of lifestyle advice

The results of multilevel regression models showed some factors influencing PPLA. For personal characteristics in Model 1, females; those who had lower education level; those with weaker social support; and individuals with higher self-reported health inside the family were significantly less likely to perceive lifestyle advice. These factors remained consistent in models 2 and 3, where we entered more factors into the model. Our results also suggested that patients with HS who took any treatments or who had a comorbidity had a significantly higher possibility of perceived lifestyle advice. Furthermore, patients who utilised family doctors’ services following HS were more likely to report PPLA (**Table 3**).

**Table 3:** Multilevel regression results of factors influencing patients’ perception of lifestyle advice

	Hypertension HS, OR (SE)						Diabetes HS, OR (SE)					
**Variables**	**Model 1**	***P*-value**	**Model 2**	***P*-value**	**Model 3**	***P*-value**	**Model 1**	***P*-value**	**Model 2**	***P*-value**	**Model 3**	***P*-value**
Age	1.217 (0.070)	0.903	1.040 (0.087)	0.638	1.010 (0.087)	0.904	1.203 (0.096)	0.202	0.875 (0.099)	0.237	0.875 (0.100)	0.242
Sex	0.887 (0.055)	<0.001	0.730 (0.066)	<0.001	0.709 (0.066)	<0.001	0.221 (0.107)	0.023	0.317 (0.176)	0.040	0.273 (0.176)	0.018
Han population	1.007 (0.135)	0.959	0.908 (0.188)	0.641	0.848 (0.183)	0.447	1.134 (0.203)	0.482	1.180 (0.334)	0.558	1.181 (0.351)	0.575
Married	0.735 (0.074)	0.792	0.810 (0.111)	0.124	0.822 (0.114)	0.156	0.749 (0.112)	0.054	0.819 (0.164)	0.319	0.903 (0.188)	0.622
Education level	1.101 (0.040)	<0.001	1.141 (0.058)	<0.001	1.140 (0.061)	0.014	1.128 (0.050)	<0.001	1.129 (0.071)	0.044	1.122 (0.072)	0.024
Occupation	1.003 (0.038)	0.931	0.941 (0.048)	0.238	0.937 (0.049)	0.211	0.942 (0.042)	0.173	0.870 (0.060)	0.106	0.865 (0.060)	0.306
Residence	1.132 (0.121)	0.248	1.023 (0.154)	0.878	1.062 (0.165)	0.699	0.870 (0.111)	0.277	0.733 (0.141)	0.210	0.751 (0.150)	0.152
Health insurance type	1.015 (0.130)	0.910	1.100 (0.181)	0.564	1.086 (0.181)	0.618	1.490 (0.208)	0.401	1.787 (0.411)	0.102	1.769 (0.413)	0.104
Household expenditure	0.993 (0.049)	0.889	1.087 (0.070)	0.194	1.112 (0.072)	0.070	0.944 (0.066)	0.413	0.950 (0.083)	0.560	0.981 (0.089)	0.830
Social support inside the family	1.473 (0.062)	<0.001	1.243 (0.066)	<0.001	1.236 (0.067)	<0.001	1.632 (0.112)	<0.001	1.349 (0.099)	<0.001	1.303 (0.099)	<0.001
Social support outside the family	1.057 (0.032)	0.002	1.082 (0.041)	<0.001	1.092 (0.043)	0.026	1.038 (0.040)	0.002	1.114 (0.053)	0.023	1.134 (0.054)	<0.001
Self-reported health status	1.251 (0.065)	<0.001	1.243 (0.081)	<0.001	1.203 (0.082)	<0.001	1.334 (0.105)	<0.001	1.297 (0.128)	<0.001	1.289 (0.133)	0.013
Activities of daily living	1.074 (0.019)	0.873	0.998 (0.026)	0.937	1.011 (0.026)	0.684	1.076 (0.027)	0.623	0.986 (0.035)	0.695	0.994 (0.039)	0.879
Regional location	0.986 (0.056)	0.810	0.952 (0.071)	0.509	0.975 (0.076)	0.747	0.935 (0.061)	0.301	0.879 (0.081)	0.164	0.896 (0.085)	0.245
Provincial GDP	1.032 (0.067)	0.625	1.047 (0.073)	0.509	1.075 (0.079)	0.324	1.046 (0.075)	0.532	1.110 (0.096)	0.227	1.126 (0.100)	0.181
Treatment regimes			1.755 (0.086)	<0.001	1.741 (0.089)	<0.001			1.761 (0.096)	<0.001	1.722 (0.094)	<0.001
Comorbidity			3.927 (0.382)	<0.001	3.979 (0.403)	<0.001			3.432 (0.302)	<0.001	3.523 (0.317)	<0.001
Utilisation of family doctors					2.699 (0.668)	<0.001					1.688 (0.570)	<0.001
Satisfaction with health care					0.852 (0.056)	0.120					0.872 (0.079)	0.131
Constant	1.413 (0.080)	0.025	1.305 (0.098)	<0.001	1.348 (0.109)	<0.001	1.327 (0.090)	<0.001	1.262 (0.090)	<0.001	1.262 (0.090)	<0.001

GDP – gross domestic product, HS – health shocks, OR – odds ratio, SE – standard error

### The association between HS, PPLA, and HB

#### The direct effect of HS on preventive behaviours

Our mediation models showed a significant direct effect of HS on HB (**Table 4**), while controlling for other variables (**Table 1**). Specifically, we found a significant decrease in smoking and drinking and an increase in exercise in both the hypertension and diabetes HS following a HS.

**Table 4:** Mediation model results of patients’ perception of lifestyle advice*

	Hypertension HS, coefficient (SE)								Diabetes HS, coefficient (SE)							
**Advice and behaviours**	**HS-HB**	***P*-value**	**HS-PPLA**	***P*-value**	**HS PPLA-HB**	***P*-value**	**Sobel test**	***P*-value**	**HS-HB**	***P*-value**	**HS-PPLA**	***P*-value**	**HS PPLA-HB**	***P*-value**	**Sobel test**	***P*-value**
Any lifestyle advice																
*Smoking*	−0.023 (0.008)	< 0.001	0.121 (0.004)	< 0.001	−0.003 (0.015)	0.827	−0.001 (0.001)	0.826	−0.037 (0.011)	0.001	0.171 (0.004)	< 0.001	0.031 (0.023)	0.175	0.005 (0.003)	0.175
*Drinking*	−0.006 (0.009)	0.061	0.121 (0.004)	< 0.001	−0.002 (0.017)	0.890	−0.001 (0.002)	0.889	−0.029 (0.012)	0.015	0.171 (0.004)	< 0.001	−0.032 (0.025)	0.196	−0.005 (0.004)	0.196
*Exercise*	0.016 (0.012)	0.081	0.194 (0.006)	< 0.001	0.096 (0.018)	< 0.001	0.018 (0.003)	< 0.001	0.056 (0.015)	< 0.001	0.256 (0.005)	< 0.001	0.088 (0.027)	0.001	0.023 (0.007)	0.001
Smoking cessation advice																
*Smoking*	−0.018 (0.008)	0.084	0.064 (0.003)	< 0.001	−0.094 (0.020)	< 0.001	−0.006 (0.001)	0.021	−0.034 (0.012)	0.004	0.075 (0.003)	< 0.001	−0.113 (0.031)	< 0.001	−0.008 (0.002)	< 0.001
*Drinking*	−0.006 (0.009)	0.051	0.064 (0.003)	< 0.001	0.001 (0.021)	0.980	−0.001 (0.010)	0.979	−0.023 (0.013)	0.066	0.075 (0.003)	0.001	−0.006 (0.034)	0.849	−0.000 (0.002)	0.849
*Exercise*	0.080 (0.012)	0.062	0.107 (0.005)	< 0.001	0.104 (0.023)	< 0.001	0.011 (0.002)	< 0.001	0.052 (0.016)	0.002	0.118 (0.004)	< 0.001	0.131 (0.036)	< 0.001	0.015 (0.004)	0.001
Dietary advice																
*Smoking*	−0.025 (0.008)	0.003	0.099 (0.004)	< 0.001	−0.023 (0.017)	0.164	−0.002 (0.001)	0.164	−0.017 (0.006)	0.001	0.158 (0.004)	< 0.001	0.016 (0.023)	0.487	0.004 (0.003)	0.487
*Drinking*	−0.005 (0.009)	0.051	0.009 (0.004)	< 0.001	−0.001 (0.018)	0.050	−0.001 (0.011)	0.051	−0.030 (0.012)	0.001	0.158 (0.004)	< 0.001	−0.037 (0.025)	0.196	−0.002 (0.006)	0.041
*Exercise*	0.015 (0.012)	0.062	0.161 (0.005)	< 0.001	0.105 (0.019)	< 0.001	0.017 (0.003)	< 0.001	0.057 (0.015)	< 0.001	0.239 (0.005)	< 0.001	0.095 (0.028)	0.001	0.023 (0.006)	< 0.001
Physical activity advice																
*Smoking*	−0.023 (0.008)	0.071	0.077 (0.003)	0.082	−0.021 (0.018)	0.235	−0.001 (0.001)	0.236	−0.045 (0.011)	0.001	0.117 (0.003)	< 0.001	0.008 (0.026)	0.749	0.001 (0.003)	0.749
*Drinking*	−0.006 (0.009)	0.456	0.077 (0.003)	< 0.001	−0.009 (0.019)	0.623	−0.001 (0.001)	0.623	−0.028 (0.012)	0.001	0.117 (0.003)	< 0.001	−0.028 (0.028)	0.328	−0.003 (0.003)	0.328
*Exercise*	0.014 (0.012)	0.052	0.128 (0.005)	< 0.001	0.140 (0.021)	< 0.001	0.018 (0.002)	< 0.001	0.066 (0.016)	< 0.001	0.182 (0.005)	< 0.001	0.168 (0.030)	< 0.001	0.030 (0.005)	< 0.001

HS – health shocks, PPLA – patients’ perception of lifestyle advice, HB – health behaviours

*All the mediation models controlled 15 variables listed in **Table 1**.

#### The positive association between HS and PPLA

The coefficients in the HS-PPLA indicated a positive association between PPLA and HS (**Table 4**). Following hypertension HS or diabetes HS, individuals were more likely to report receiving lifestyle advice from health care providers; this association was statistically significant. We observed a similar pattern for smoking cessation, dietary, and physical activity advice, showing a positive correlation with HS in each case.

#### The mediating effect of PPLA

We observed an indirect effect after including PPLA as a mediator in the models, suggesting that lifestyle advice with any contents mediates the relationship between HS and exercise. The significance of Sobel tests provided evidence for the validation of the mediation effects of PPLA. We did not examine this mediating role of PPLA without tailored contents in the relationship between HS and smoking or drinking.

#### The mediating effect of different contents of PPLA

The results regarding the mediating role of different lifestyle advice contents demonstrated that targeted and detailed lifestyle advice mediated the impact of HS on corresponding addictive HB, such as smoking and drinking. In terms of smoking, the mediating role of PPLA existed only when individuals with HS perceived smoking cessation advice, leading to a decrease in smoking. Regarding drinking, patients decreased drinking when they received dietary advice, and PPLA acted as a significant mediator. Simultaneously, the mediation effect remained unchanged for non-addictive behaviour (exercise). Regardless of the specific content of the lifestyle advice, individuals who perceived any advice exhibited increased exercise, and the mediation effect of PPLA was significant.

## DISCUSSION

In line with previous research, we found that HS has a positive impact on HB, with a significant decrease in smoking and drinking, and an increase in exercise [9–14]. As far as we know, this is the first study to examine the mechanism of HS promoting HB and to prove the mediating role of PPLA.

Our results confirm that HS can serve as an educational moment and that the mediation effect of PPLA between HS and non-addictive HB, exercise, remained unchanged irrespective of the content of the advice. By increasing health care utilisation and the intensity of doctor-patient contact after a HS [11,12,17,18,20–22], HS bring an opportunity for health care professionals to educate patients and provide more acceptable, persuasive lifestyle advice [45]. Patients at an early stage of newly diagnosed chronic disease are also more susceptible and convincible to health education, leading to a higher compliance with lifestyle advice, which eventually results in taking HB, in turn leading to more effective promotion of HB and prevention of complications [23–27].

Notably, only targeted and detailed lifestyle advice mediates the effect of HS on corresponding addictive HB (smoking, drinking). Patients with addictive behaviours are more likely to have resistant attitudes to accept preventive suggestions. Also, the broad and indirect advice without targeted content has a limited specific impact on addictive behaviours with long-term behavioural inertia. Additionally, heavy smokers or alcoholics may be physiologically unable to quit merely by inaccurate advice, calling for more supportive assistance and detailed arrangements, like the nicotine patch or Alcoholics Anonymous meetings [46].

We found limited and selective reception of lifestyle advice in our study. Despite the clinical guidelines [34,35] and compulsive requirements in practice for providers proposing lifestyle advice, merely half of patients with HS reported receiving advice. The limited reception suggests a gap between the advice given by providers and the advice perceived by patients, which reflects that the latter cannot obtain, understand, and remember all lifestyle advice, health information, and interventions [37]. There is room to utilise the potential for education of the HS to improve health awareness and behaviours. Also, our findings show that patients selectively perceived different contents of advice, with smoking cessation advice having the lowest acceptance rate compared to dietary advice and physical activity advice. Like previous studies reporting low acceptance of smoking cessation advice, patients with the most addictive behaviour have the greatest difficulty in perceiving advice or changing behaviours, highlighting that PPLA rather than the provision of advice can turn interventions into HB [36,37].

Furthermore, we explored the factors influencing PPLA and explored the potential reason for limited perception. Education level and social support within the family serve as positive factors, suggesting that patients’ capability of understanding and social resources may help them perceive health information [47,48]. Worse self-reported health and comorbidity elevate personal awareness of health risks, making patients more receptive to advice and interventions [23–27]. Treatment regimens and utilisation of family doctors were positively associated with PPLA, demonstrating the importance of increasing doctor-patient contact in promoting HB [17,18,20–22].

This study has important policy implications in the context of China, as it had extensively implemented family doctor contract services in recent years, thus providing personalised management for chronic diseases. The gap between the provision and the perception of advice highlights the need for more targeted and personalised preventive interventions. Healthcare providers should seize the opportunity of educational opportunity and offer patient-centred care, especially by offering specific and tailored advice. By integrating relevant policies of poverty alleviation in China, efforts are made to provide stronger social support and improve health literacy for disadvantaged populations.

Several limitations should be considered in interpreting our results. First, we used nationally representative longitudinal data from CHARLS; however, the focus of this single on the middle- and older-aged Chinese population and its limited data on follow-up visits leads to potential for selection bias; constraints in generalisability to other age groups or populations in different geographic or cultural contexts; and a lack of long-term observation. Second, the provision of lifestyle advice was not objectively measured using individual survey data, but rather through self-reporting, which may have caused an overestimation of advice provision. However, patient self-report is considered the most credible and relatively objective measure of advice provision compared to doctor reports [36,49]. Third, although we employed the PSM method to construct two well-balanced groups at baseline, there may still be unobservable factors that contribute to differences between the groups, including individual health beliefs, risk preferences, and other psychological factors.

We suggest that future independent studies should be designed to record real-time and objective advice provision during scenarios such as doctor visits and compare it with perceived advice from patients to identify any differences. There is also a need for long-term observation of HB changes, particularly in exploring the longitudinal impact of HS on HB and the effectiveness of different types of lifestyle advice based on experimental or quasi-experimental designs.

## CONCLUSIONS

This study contributes to the current literature by testing a mechanism between HS and HB. We found limited and selective PPLA, analysed its influencing factors, and proved it acts as a general partial mediator for HS on non-addictive HB and a specific partial mediator of tailored content for HS on corresponding addictive HB. Personalised chronic disease management, targeted advice and interventions, and multiple social resources may prove beneficial for patients with HS in perceiving lifestyle advice and initiating HB.
